# Whole genome sequence of a *Mycobacterium tuberculosis* M64 isolate harboring extensive deletions, including the *katG*-containing region

**DOI:** 10.1128/mra.00745-24

**Published:** 2024-12-10

**Authors:** Se-Mi Jeon, Sanghee Park, Seonghan Kim

**Affiliations:** 1 Department of Bacterial Disease Research, Center for Infectious Disease Research, National Institute of Infectious Disease, National Institute of Health, Korea Disease Control and Prevention Agency, Cheongju-si, South Korea; Department of Biological Sciences, Wellesley College, Wellesley, Massachusetts, USA

**Keywords:** *Mycobacterium tuberculosis*, resistance, isoniazid, *katG*

## Abstract

Clinical isolates of *Mycobacterium tuberculosis* M64 strain from South Korea lack the *katG* gene, which is associated with isoniazid resistance. Herein, we report the whole genome sequence of *M. tuberculosis* M64, with genomic coverage of 2,791× and a genome size of 4,353,699 bp.

## ANNOUNCEMENT

*Mycobacterium tuberculosis* continues to be a global health issue that has resulted in approximately 1.3 million deaths (1). Tuberculosis treatment regimens typically comprise four first-line anti-tuberculosis drugs, namely rifampicin, isoniazid, pyrazinamide, and ethambutol, that are administered over 6–9 months (2). Long-term treatment, relapse, and adverse effects are the primary causes of treatment failure (3, 4).

We analyzed *M. tuberculosis* M64 isolated from a patient admitted to the Masan National Tuberculosis Hospital in 2011. The patient underwent long-term treatment and exhibited multidrug resistance. The study was approved by the Korea Disease Control and Prevention Agency (KDCA) Institutional Review Board 2022-08-02-C-A and the KDCA Institutional Biosafety Committee KDCA-IBC-2022-041.

*M. tuberculosis* isolated from sputum was incubated in 7H9 broth containing 0.02% Tween 80 and 0.5% glycerol at 37°C with shaking at 180 rpm for 10–14 days. Genomic DNA was extracted using a Quick-DNA Fungal/Bacterial Kit (Zymo Research, Irvine, CA, USA) following the manufacturer’s instructions (5). TapeStation DNA ScreenTape (Agilent, USA) and TruSeq Nano DNA Sample Prep Kit (Illumina, San Diego, CA, USA) were used for sample QC and library preparation, respectively. Deep sequencing was performed using paired-end reads of 2 × 250 bp on a NovaSeq 6000 (Illumina). Re-sequencing was analyzed using the sequence with the accession number NC_000962.3 of the reference strain *M. tuberculosis* H37Rv ATCC 27294. Trimming and assembly were performed using the CLC Genomics Workbench v22 (Qiagen, Germantown, MD, USA), and the quality and validity of the final genome assembly were evaluated using the quality control tool of the CLC Genomics Workbench.

The sequencing output included reads spanning a total of 89,688,290 bp with a genomic coverage of 2,791×. The genome size was 4,353,699 bp, with a Guanine-Cytosine (GC) content of 65.5%. The whole genome sequencing (WGS) results were submitted to NCBI for annotation analysis using the NCBI Prokaryotic Genome Annotation Pipeline (v6.7) (6). Annotations made with reference to the sequence NC_000962.3 of *M. tuberculosis* H37Rv ATCC 27294 revealed the absence of the *katG*-containing region between genomic positions 2,151,623 and 2,166,082 (14,459 bp), which is associated with isoniazid resistance.

## Data Availability

The sequenced genomic data have been deposited in DDBJ/EMBL/GenBank under accession no. CP150983. The Whole Genome Shotgun project is available in the BioSample accession no. PRJNA1092334 and SRA accession no. SRR28467020.
